# EXAMINING GENDER DIFFERENCES IN RELIGIOUS INVOLVEMENT AND DEPRESSION IN LATER LIFE

**DOI:** 10.1093/geroni/igz038.1933

**Published:** 2019-11-08

**Authors:** Hailey J Santiago, Caitlin Curtin, Julia Stengel, Edward H Thompson, Andrew Futterman

**Affiliations:** 1 Loyola University Maryland, Baltimore, Maryland, United States; 2 College of the Holy Cross, Worcester, Massachusetts, United States; 3 Department of Psychology, Loyola University Maryland, Baltimore, Maryland, United States

## Abstract

This study examines gender differences in a causal model of religious motivation, religious participation and depression. Using a random sample of 287 community-dwelling older adults living in Worcester, MA, the model hypothesizes that motivations for religious involvement (intrinsic vs. extrinsic) differentially predict religious participation (organizational and non-organizational) as well as depression at both initial and 12-month assessments. In this model, participation also mediates direct relationships between religious motivation and depression. Religious motivation and participation are assessed using standard measures (e.g., Allport & Ross, 1967; Ainlay & Smith, 1982), and depression is assessed both by self-report (CESD and by interview (Hamilton Rating Scale for Depression derived from the Schedule for Affective Disorders and Schizophrenia, SADS). Using MPlus, confirmatory analyses of the model were conducted separately in male and female samples. The model which includes both direct effects of religious motivation and participation on depression and with religious participation as mediating variable demonstrated reasonably good fit to the data in both male and female samples (e.g., CFI=.956 and .943, respectively). Consistent with previous research (e.g., McFarland, 2009), gender differences in the models emerge. For example, men report higher levels of religious participation and less depression than women. In addition, older men demonstrate stronger positive associations between extrinsic religiousness and organizational participation and a more negative association between extrinsic religiousness and depression, than older women. Elucidating the structural relationships among religious orientation, religious participation, and depression in older adults benefits our understanding of vulnerability and treatment of depression in this population.

